# Association of prenatal sex steroid exposure estimated by the digit ratio (2D:4D) with birth weight, BMI and muscle strength in 6- to 13-year-old Polish children

**DOI:** 10.1371/journal.pone.0258179

**Published:** 2021-10-04

**Authors:** Magdalena Kobus, Aneta Sitek, Iwona Rosset, Paulina Pruszkowska–Przybylska, Elżbieta Żądzińska

**Affiliations:** 1 Department of Anthropology, Faculty of Biology and Environmental Protection, University of Lodz, Lodz, Poland; 2 Biological Anthropology and Comparative Anatomy Research Unit, School of Medicine, University of Adelaide, Adelaide, South Australia, Australia; University of Cambridge, UNITED KINGDOM

## Abstract

**Objectives:**

The aim of this paper was to provide evidence for the impact of prenatal sex steroid exposure on prenatal and postnatal body size parameters, and muscle strength in children.

**Methods:**

The following anthropometric data were studied in a group of 1148 children (536 boys and 612 girls) aged 6–13 years: the 2D:4D digit ratio, birth weight and length, and birth head and chest circumference. Postnatal parameters (6–13 years) included body weight and height, BMI, waist and hip circumference, WHR, as well as grip strength in both hands. All parameters that required it were adjusted for sex and gestational or chronological age. A general linear model, Pearson’s correlation, *t*-statistics and Cohen’s Δ were used in statistical analysis.

**Results:**

Among birth size parameters, only birth weight was significantly negatively correlated with the 2D:4D digit ratio in children. Higher (feminized) digit ratios were significantly correlated with postnatal parameters such as body weight, BMI, and waist and hip circumference (positively), as well as hand grip strength–a proxy for muscular strength (negatively).

**Conclusion:**

Problems with maintaining adequate body size parameters and muscle strength may be programmed in fetal life and predicted on the basis of the 2D:4D digit ratio. Body weight at birth and in early ontogenesis are additive correlates of the 2D:4D ratio. The present findings suggest that the 2D:4D digit ratio is related to postnatal phenotypes such as birth weight, overweight, and obesity as well as muscle strength in 6–13-year-old children of both sexes.

## Introduction

The prenatal period is crucial for child development and any interfering conditions may disturb all subsequent ontogenesis. Sex steroid exposure during fetal life seems to be a significant factor shaping later stages of development [1, 2]. Evaluation of the second-to-fourth digit length ratio (2D:4D) in the human hand is a widely known method of assessing the proportions of prenatal sex steroids [3, 4].

Evidence shows that the digit ratio remains stable throughout ontogenesis, being established in the second trimester of pregnancy. Males typically have lower ratios (with longer fourth fingers) than females (with shorter fourth fingers). A 2011 study by Zheng and Cohn [5] confirmed the hypothesis that 2D:4D results from sex-related differences in testosterone and estrogen levels during pregnancy. Their experiments on mice proved that digit growth is affected by the activity of androgen and estrogen receptors. In 1998 Manning et al. reported for the first time that the right hand is more sensitive to prenatal levels of sex steroids compared to the left hand [5]. Further studies also suggested that right hand is better indicator of prenatal sex steroid exposure [6, 7]. The present study shows that 2D:4D can be used as an indicator of dimorphism and provides empirical evidence that it can reflect prenatal hormone levels.

To date few studies have analyzed the 2D:4D digit ratio and body parameters or proportions in adults, with the findings remaining inconclusive [8–12]. However, recently published study conducted by Manning et al. showed that 2D:4D is positively related to BMI in a very large sample of adults (255,116 participants) [13]. This indicates the need to investigate whether and how prenatal sex steroid exposure affects developmental processes from prenatal stages through childhood and adolescence to adulthood. In the literature there are only a few papers describing significant relationships between 2D:4D and body size parameters in children and adolescents [12, 14, 15], while some authors have reported no correlations for those groups [16–18]. Analysis of this phenomenon at the level of early ontogenesis requires more data on children, and so the present study makes an important contribution to this field.

The main objective of this investigation was to establish whether sex steroid proportions during pregnancy have a long-lasting effect and are involved in determining birth size as well as body parameters and muscle strength in mostly prepubertal children.

## Material and methods

Analysis involved a database containing 1148 records for children aged 6–13 years: 536 boys and 612 girls from central Poland. Participation in the study was conditional on written consent by the parents, who also completed the questionnaires. The study was approved by the Ethical Committee of the University of Lodz (no. 19/KBBN-UŁ/II/2016). Data were collected between 2016 and 2018.

Children were recruited at primary schools in Lodz, a city with 677,000 inhabitants located in the center of Poland. Information about birth parameters, i.e., birth weight and length, head and chest circumference, and gestational age (week of pregnancy) were noted by physicians immediately after birth and taken from children’s medical records. Further data were collected by anthropometric measurements according to Martin’s standard procedures [19]. Postnatal somatometric measurements were performed by professional staff of the Department of Anthropology University of Lodz when children were 6 to 13 years old. Measurements included: body weight and height, waist and hip circumference, length of the second and fourth digits, and hand grip strength. The following tools were used: an electronic scale (body weight), an anthropometer (height), an anthropometric tape measure (circumferences), a sliding Vernier caliper (length of the second and four digits), and a DRP 90 flat-spring dynamometer (hand grip strength). 2D and 4D lengths were taken on the palmar surface of the outstretched right and left hands. Waist circumference was measured at the narrowest part of the torso and hip circumference was taken at the widest part of the hip with a tape measure. The body mass index (BMI) was calculated as weight in kilograms divided by the square of height in meters. The waist-to-hip (WHR) ratio was computed by dividing waist circumference (cm) by hip circumference (cm). The muscle strength of each hand was calculated as the average value of three squeezes in dynamometric tests.

### Statistical analyses

A general linear model (GLM) was used to assess the significance of the main effects and interactions between factors to explain the variability of individual dependent variables (Statistica PL.13.0 software). The relationship between 2D:4D indicators in girls and boys is illustrated in graphs with individual dependent variables. This relationship was evaluated using Pearson’s linear correlation coefficient (*r*), with its significance assessed by *t-*statistics (Statistica PL.13.0 software). Cohen’s effect sizes (Cohen’s Δ) were calculated using Effect Size Calculator for T-Test (https://www.socscistatistics.com/effectsize/default3.aspx).

## Results

Incomplete records were not excluded from the study, and so population size differed depending on the analyzed feature (Table 1). Effect sizes (Cohen’s Δ) were calculated to quantify the magnitude of the sex differences in the parameters (Table 2). All analysed prenatal and postnatal body size parameters were significantly higher in boys than in girls, although the effect size for sex differences was rather small (Cohen’s Δ about 0.3).

**Table 1:** Characteristics of body size parameters.

Parameter		Sex	N	Mean	SD	Median	Q25	Q75
**PRENATAL STAGE (BIRTH)**	Birth weight	Boys	536	3372.38	535.96	3370.00	3100.00	3700.00
		Girls	612	3218.84	490.98	3250.00	2950.00	3500.00
		Total	1148	3290.72	518.02	3300.00	3000.00	3600.00
	Birth length	Boys	525	54.64	3.98	55.00	53.00	57.00
		Girls	601	53.78	3.21	54.00	52.00	56.00
		Total	1126	54.18	3.61	54.00	52.00	56.00
	Head circumference	Boys	472	34.39	1.78	34.00	34.00	36.00
		Girls	547	33.75	1.57	34.00	33.00	35.00
		Total	1019	34.05	1.70	34.00	33.00	35.00
	Chest circumference	Boys	461	33.41	2.31	34.00	32.00	35.00
		Girls	539	32.87	1.99	33.00	32.00	34.00
		Total	1000	33.12	2.16	33.00	32.00	34.00
**POSTNATAL STAGE**	Body weight	Boys	602	35.33	11.50	32.65	27.05	40.95
		Girls	664	32.13	10.22	29.70	24.80	36.70
		Total	1266	33.65	10.96	31.00	25.70	39.00
	Body height	Boys	601	137.75	11.96	136.70	129.00	144.70
		Girls	664	134.96	11.69	134.00	126.50	141.70
		Total	1265	136.29	11.90	135.20	127.90	143.40
	BMI	Boys	601	18.23	3.55	17.22	15.70	20.02
		Girls	664	17.26	3.10	16.47	15.08	18.62
		Total	1265	17.72	3.36	16.79	15.35	19.39
	Waist circumference	Boys	601	64.42	10.28	61.00	57.00	70.00
		Girls	664	60.42	9.08	58.00	54.00	64.00
		Total	1265	62.32	9.87	60.00	55.00	67.50
	Hip circumference	Boys	600	74.14	9.88	72.50	67.00	81.00
		Girls	663	72.38	9.48	71.00	65.00	78.00
		Total	1263	73.21	9.71	72.00	66.00	79.50
	WHR	Boys	600	0.87	0.06	0.86	0.83	0.91
		Girls	663	0.84	0.06	0.84	0.80	0.87
		Total	1263	0.85	0.06	0.85	0.81	0.89
	R-HGS	Boys	258	11.48	4.73	11.00	8.00	14.00
		Girls	286	9.63	4.06	9.00	6.50	12.00
		Total	544	10.51	4.48	10.00	7.00	13.00
	L-HGS	Boys	258	10.35	4.42	10.00	7.00	12.03
		Girls	286	8.95	3.79	9.00	6.00	11.00
		Total	544	9.61	4.16	9.00	6.50	11.50

**Table 2:** Body size parameters and Cohen’s effect sizes.

	Parameter	t	p	Cohen’s Δ
**PRENATAL STAGE (BIRTH)**	Birth weight^1^	-7.48	<0.01	0.33
	Birth length	-5.83	<0.01	0.03
	Head circumference^1^	-6.36	<0.01	0.03
	Chest circumference^1^	-9.34	<0.01	0.44
**POSTNATAL STAGE**	Body weight^1^^.^^2^	-6.79	<0.01	0.18
	Body height^2^	-5.64	<0.01	0.15
	BMI^1^^.^^2^	-5.71	<0.01	0.15
	Waist circumference^1^^.^^2^	-6.4	<0.01	0.18
	Hip circumference^1^^.^^2^	-2.3	0.02	0.07
	WHR^1^^.^^2^	-10.49	<0.01	0.33
	R-HGS^1^^.^^2^	-9.13	<0.01	0.31
	L-HGS^1^^.^^2^	-8.24	<0.01	0.27

^1^After Box-Cox transformation.

^2^Adjusted for chronological age.

Repeated measurements of the length of the second and fourth digits in 30 hands were taken. Intra-class correlations between these measurements were high and ranged from 0.97 to 0.99 (2D R: r = 0.99, 4D R: r = 0.98, 2D R: = 0.99, 4D L: r = 0.97). Thus, the measurements of digit lengths were highly accurate and repeatable. Table 3 presents descriptive statistics of 2D:4D R & L in boys and girls. 2D: 4D digit ratios showed dimorphic differences in children (2D:4D R: t = -5.46, p < 0.01; 2D:4D L: t = -4.52, p< 0.01) and their direction were as expected (girls had higher mean of 2D:4D R & L than boys).

**Table 3:** Characteristics of 2D:4D in boys and girls and Cohen’s effect sizes.

2D:4D	Sex	n	x	SD	t	P	Cohen’s Δ
2D:4D R	Boys	602	0.974	0.034	5.46	<0.01	0.31
	Girls	664	0.984	0.032			
2D:4D L	Boys	602	0.973	0.031	4.52	<0.01	0.25
	Girls	664	0.981	0.030			

Statistical analysis showed that among birth size parameters only birth weight (adjusted for sex and gestational age) was significantly negatively correlated with right (but not left) 2D:4D, which means that feminized 2D:4D R ratios were associated with lower birth weight than that expected for sex and gestational age. As the interaction of 2D:4D R with sex was not significant (*p* = 0.41), the direction of this correlation applied both to girls and boys (Table 4).

**Table 4:** Associations of right and left digit ratios (2D:4D R. 2D:4D L) and their interactions with sex with birth size parameters postnatal somatometric variables and grip strength of both hands.

Ontogenesis stage (measurement time)	Dependent variable	Explanatory variable^a^ 2D:4D R		Interaction^b^ 2D:4D R × sex		Explanatory variable^a^ 2D:4D L		Interaction^b^ 2D:4D L × sex	
		β	*p*	*F*	*p*	β	*p*	*F*	*p*
Prenatal stage (birth)	Birth weight^1^^.^ ^3^	**-0.07**	**0.01**	0.68	0.41	-0.05	0.08	0.03	0.85
	Birth length^1^	-0.04	0.15	0.59	0.44	-0.02	0.49	0.04	0.83
	Head circumference^1^^.^ ^3^	-0.03	0.24	1.11	0.30	-0.04	0.16	0.48	0.49
	Chest circumference^1^^.^ ^3^	-0.03	0.22	0.01	0.91	-0.03	0.30	0.04	0.84
Postnatal stage (6–13 years old)	Body weight^2^^.^ ^3^	**0.06**	**0.01**	1.74	0.19	-0.00	0.95	0.17	0.69
	Body height^2^	-0.01	0.66	1.40	0.24	-0.01	0.53	0.08	0.77
	BMI^2^^.^ ^3^	**0.08**	**<0.01**	0.85	0.36	0.01	0.77	0.38	0.54
	Waist circumference^2^^.^ ^3^	**0.05**	**0.04**	1.70	0.19	0.00	0.94	1.27	0.26
	Hip circumference^2^^.^ ^3^	**0.07**	**0.01**	3.24	0.07	0.02	0.58	1.12	0.29
	WHR^2^^.^ ^3^	0.02	0.37	0.25	0.62	-0.01	0.77	0.58	0.45
	Right hand grip strength^2^^.^ ^3^	**-0.09**	**<0.01**	1.91	0.17	**-0.08**	**<0.01**	**9.30**	**<0.01**
	Left hand grip strength^2^^.^ ^3^	**-0.06**	**0.04**	0.19	0.66	-0.05	0.11	**4.86**	**0.03**

^1^ Adjusted for sex and gestational age.

^2^ Adjusted for sex and chronological age.

^3^ After Box-Cox transformation.

^a^ Linear regression results.

^b^ General linear model (GLM) results.

β–Beta coefficient from linear regression.

*F*–Test evaluating the ratio of the variance of the predicted values to that of the residuals or error.

*p*–*p*-Value.

Most of the examined postnatal development characteristics were also associated with 2D:4D R. BMI and waist and hip circumference were positively correlated with 2D:4D R, which means that feminized digit ratios were linked to higher values of those somatometric characteristics than expected on the basis of children’s sex and age. Since the 2D:4D R–sex interaction was not significant for any variables, the direction and strength of 2D:4D R correlations with BMI and waist and hip circumference were similar in girls and boys (Table 4).

**Fig 1:**
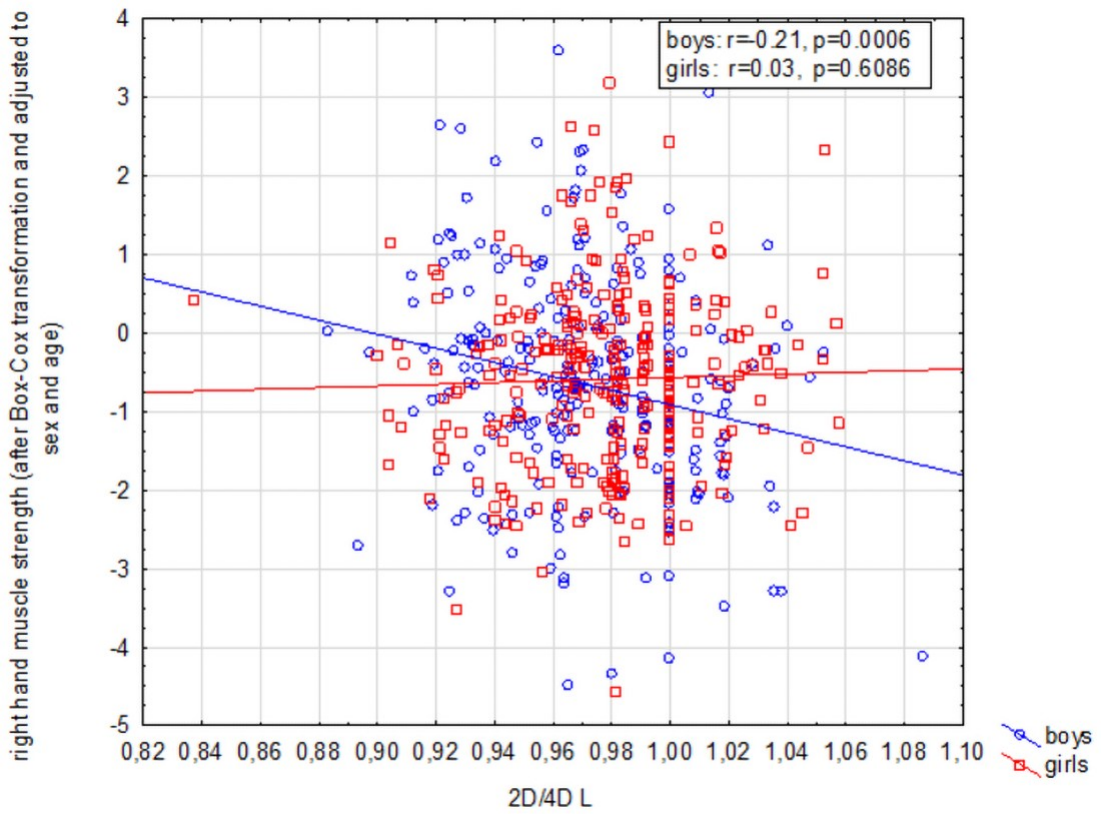
Interaction between 2D:4D L digit ratio and right hand grip strength among children aged 13–16.

**Fig 2:**
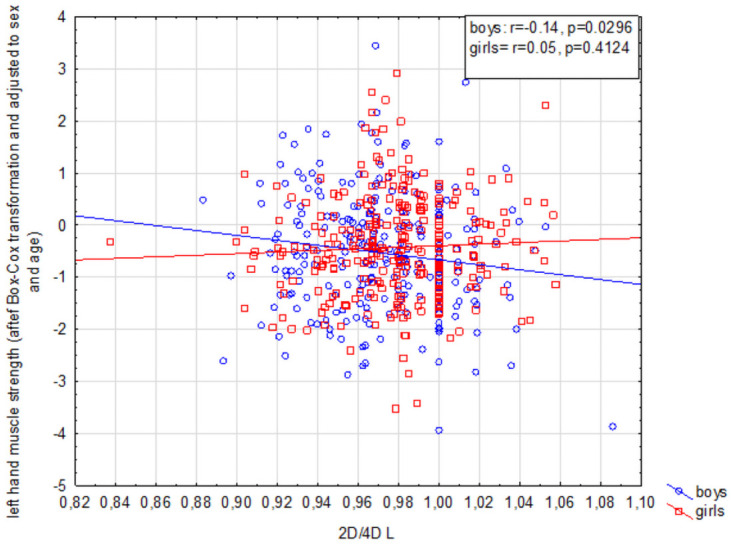
Interaction between 2D:4D L digit ratio and left hand grip strength among children aged 13–16.

Right hand grip strength was negatively correlated with the digit ratio of both hands (*p* < 0.01) and *p* < 0.01), and children’s sex did not affect this correlation for 2D:4D R (*p* = 0.17). In both groups feminized 2D:4D R values were associated with lower right hand grip strength in relation to those expected for the child’s sex and age (Fig 1). In contrast, in the case of 2D:4D L, analysis did reveal a sex-dependent relationship (*p* = 0.03) (Table 4) (Fig 2). A negative correlation between these variables was observed in boys (*r* = -0.22, *p* < 0.01), but not in girls (*r* = 0.03, *p* = 0.61) (Table 4).

2D:4D R was significantly associated with left hand grip strength as a main effect (*p* = 0.04), but children’s sex did not affect this correlation (*p* = 0.70). While 2D:4D L and the grip strength of the same hand did not exhibit covariation for the entire study group (*p* = 0.11), the association between these variables was found to be sex-dependent (*p* = 0.03) (Table 4). A negative correlation was identified in boys (*r* = -0.14, *p* = 0.03), but not in girls (*r* = 0.05, *p* = 0.42).

**Fig 3:**
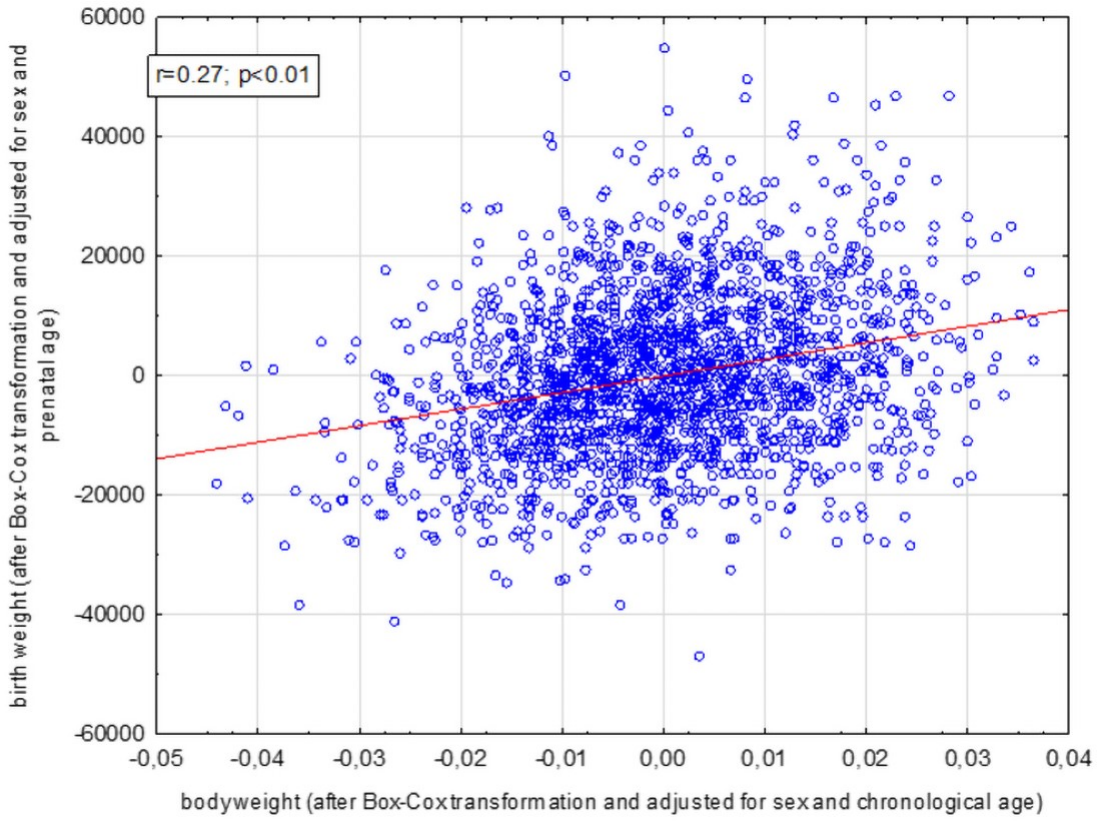
Relationship between birth weight and body weight among children aged 13–16.

Positive correlation between birth weight and postnatal body weight was found in the whole group regardless of sex (r = 0.27, p < 0.01). Higher birth weight (adjusted for gestational age and sex) was associated with higher body mass (adjusted for chronological age and sex) regardless the sex (Fig 3). Thus, this relationship was not dependent on sex steroid exposure and the next step of analysis involved its influence.

**Fig 4:**
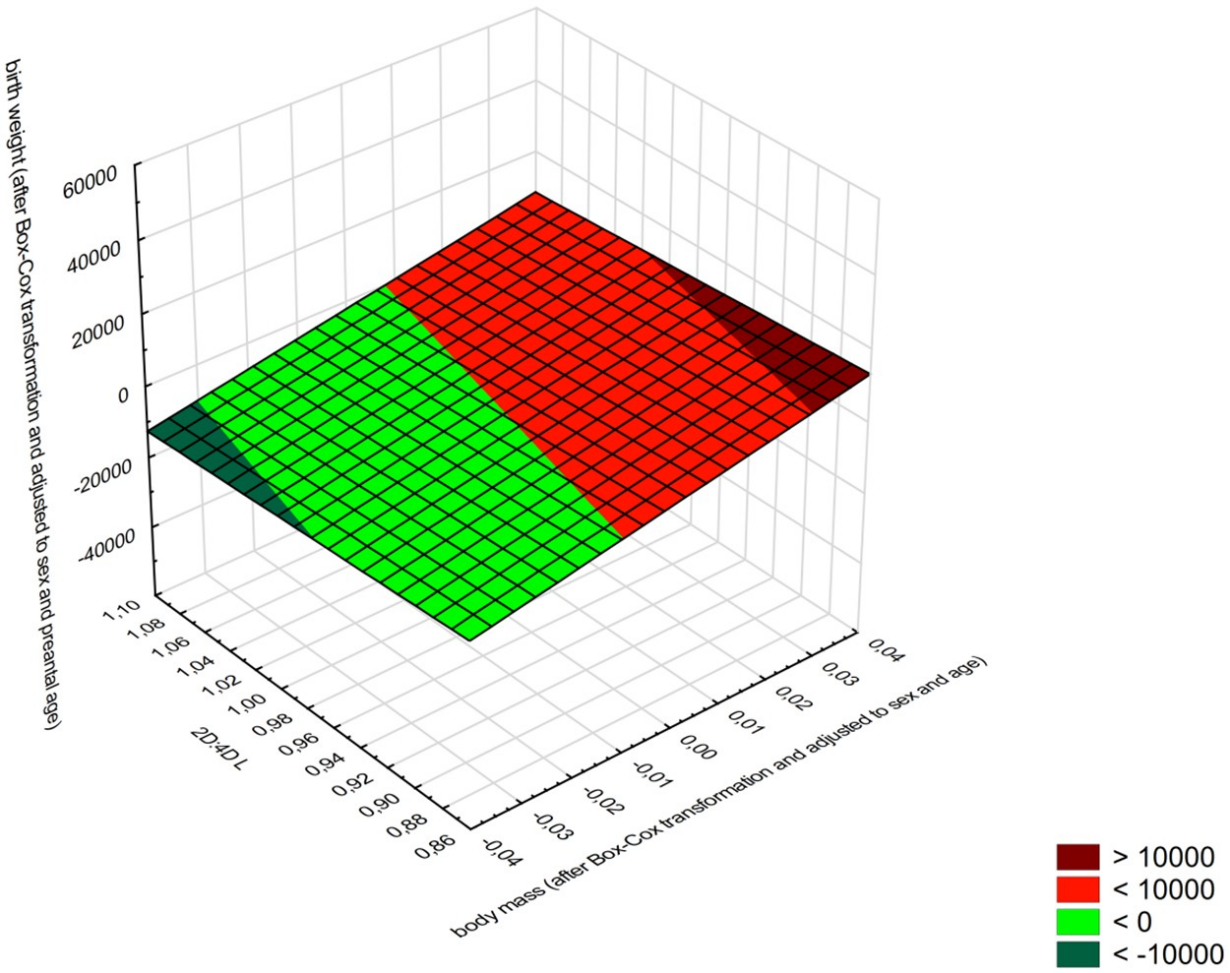
Relationship between 2D:4D L birth weight (adjusted for sex and gestational age) and body weight during postnatal ontogenesis (adjusted for sex and chronological age) in boys.

Relationship between 2D:4D R, birth weight (adjusted for sex and gestational age), and body weight during postnatal ontogenesis (adjusted for sex and chronological age) was analyzed. The objective was to establish whether children with feminized 2D:4D R and lower birth weight (compared to that expected for the child’s sex and gestational age) tended to reach specific body weight values during postnatal development. The results of individual analyses (summarized in Table 4) might suggest that in further ontogenetic stages such children may reach greater body weight than that expected for their sex and chronological age. Consequently, the interaction was evaluated separately for girls and boys and for both groups taken together. The results confirmed statistical significance only in the male group and 2D: 4D L (Table 5). Boys with feminized 2D:4D L (high prenatal oestrogen exposure) ratios and lower birth (adjusted for sex and gestational age) coexists with greater body weight in postnatal period (adjusted for sex and chronological age). However, high prenatal testosterone exposure (masculinized 2D:4D L), birth weight and postnatal body weight are positively correlated (Fig 4). In female group no such relationship with prenatal sex steroids exposure was identified. Girls with feminized 2D:4D R & L ratios and lower birth weight did not show any tendency to reach specific body weight values in postnatal development.

**Table 5:** Assessment of significance of the interaction between 2D:4D R and birth weight explaining body weight variability in children during postnatal ontogenesis.

Ontogenesis stage(measurement time)	Dependent variable	Sex	2D:4D R × birth weight^1^^.^ ^3^		2D:4D L × birth weight^1^^.^ ^3^	
			F	*p*	F	*p*
**Postnatal (6–13 years old)**	Body weight^2^^.^ ^3^	Boys	0.73	0.39	**5.31**	**0.02**
		Girls	0.06	0.80	0.09	0.77
		Total	0.00	1.00	1.08	0.30

^1^ Adjusted for sex and gestational age.

^2^ Adjusted for sex and chronological age.

^3^ After Box-Cox transformation.

The last step of analysis involved the relationship between 2D:4D R & L, birth weight (adjusted for sex and gestational age) and right hand grip strength in children during early ontogenesis (adjusted for sex and chronological age). The goal was to determine whether prepubertal children with feminized 2D:4D (R & L) and lower birth weight (than that expected for their sex and gestational age) tended to exhibit lower hand grip strength (than that expected for their sex and chronological age). The results were not statistically significant either for boys or girls separately, or for the entire group taken together (Table 6).

**Table 6:** Assessment of significance of the interaction between 2D:4D R & L and birth weight explaining right hand grip strength variability in children during postnatal ontogenesis.

Ontogenesis stage (measurement time)	Dependent variable	Sex	2D:4D R × birth weight^1^^.^ ^3^		2D:4D L × birth weight^1^^.^ ^3^	
			*F*	*p*	*F*	*p*
**Postnatal (6–13 years old)**	Right hand grip strength^2^^.^ ^3^	Boys	0.04	0.83	0.53	0.47
		Girls	1.40	0.24	1.42	0.23
		Total	0.27	0.60	0.06	0.81

^1^ Adjusted for sex and gestational age.

^2^ Adjusted for sex and chronological age.

^3^ After Box-Cox transformation.

**Fig 5:**
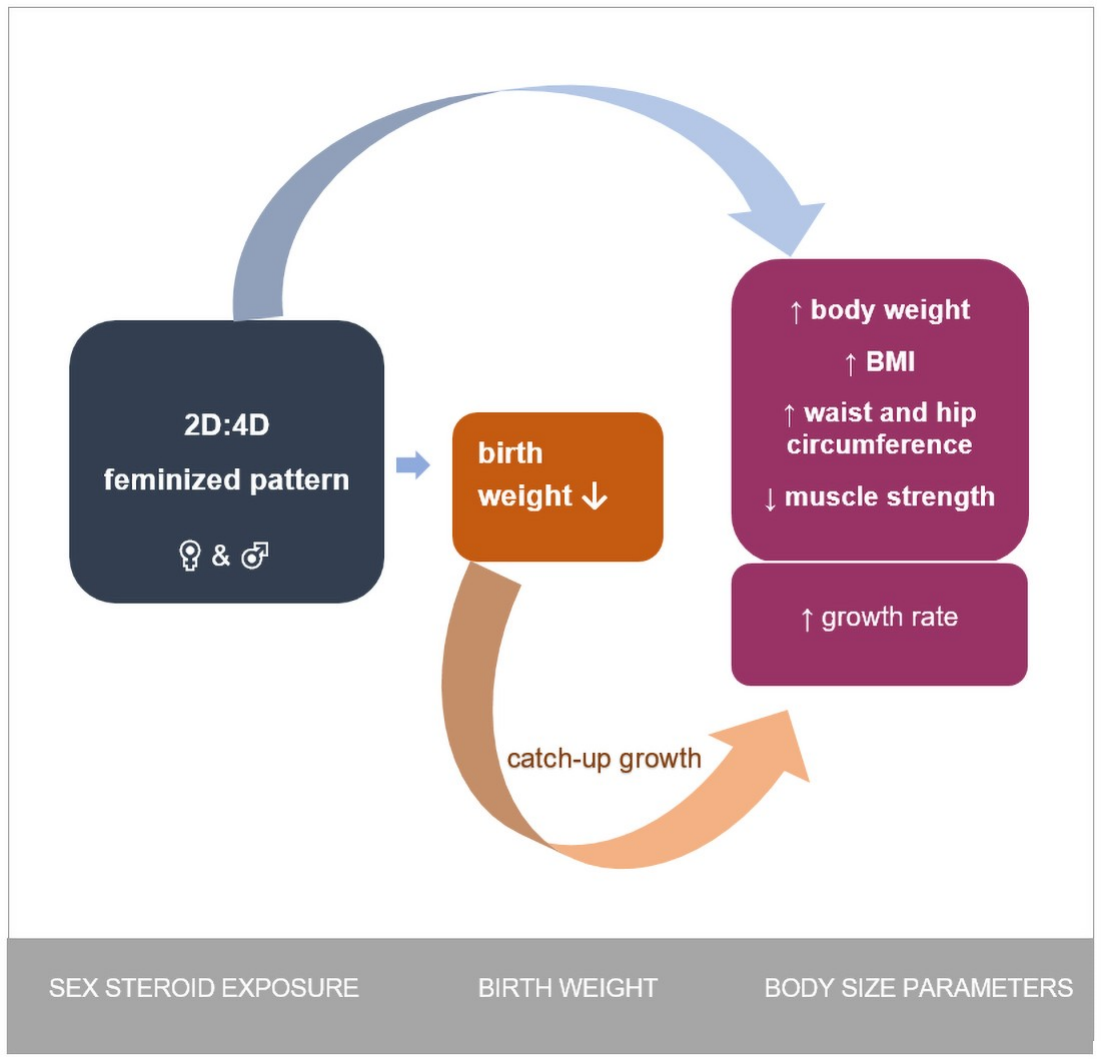
Interactions between prenatal sex steroid exposure and the processes shaping birth weight and body size parameters (blue arrows).

All interactions between variables are presented in Fig 5 (blue arrows). Additionally, a catch-up growth event is shown in the background (orange arrow).

## Discussion

Intrauterine programming plays a significant role in early ontogenesis. During critical windows. the fetus is more sensitive and any suboptimal conditions can have a range of impacts on further development stages [20–25]. For instance, metabolic and hormonal disturbances adversely influence subsequent developmental processes and are associated with an increased risk of civilization diseases, such as cardiovascular (hypertension. ischemic heart disease) and metabolic disorders (obesity, reduced tissue sensitivity to insulin, type 2 diabetes) [21, 26–28].

One of the first potential effects of prenatal development disturbances is abnormal birth weight. There are many well-known prenatal factors that affect birth weight. e.g. parental age and health, the use of stimulants during pregnancy, exposure to tobacco smoke, as well as the prenatal hormonal environment [29–32]. In the present study prenatal exposure to sex steroids was related to birth weight in children of both sexes. Feminized values of the 2D:4D digit ratio were associated with lower birth size (than those expected for the child’s sex and gestational age). Low birth weight is associated with the risk of metabolic disorders (Barker hypothesis) [26, 33]. Numerous studies have shown that low birth weight may result in increased risk of type 2 diabetes in adulthood [34–37]. It should be emphasized that the same metabolic risk also applies to high birth weight for gestational age (macrosomic newborns) [38]. In the current study, analysis was based on a large group of boys and girls (*N* = 1148). Few studies to date have reported significant correlations between 2D:4D and birth weight [16, 39]. with one paper finding no such correlation (*N* = 139) [4]. In other studies significant relationships were observed for the right hand. which is taken to be a better indicator of androgenization [5, 6]. The aforementioned publications showed different directions of correlations between the two parameters. Klimek et al. reported that men with masculinized 2D:4D R values exhibited higher birth weight (*N* = 320) [15]. While in the paper by Danborno et al. feminized 2D:4D ratios (R & L) were associated with higher birth weight in a female group (*N* = 118). Another study reported results similar to ours, but for both hands in a male group (*N* = 215), with 2D:4D (R & L) being negatively correlated with birth weight [39]. It should be noted that in the present study such a relationship was observed for boys and girls.

The current findings suggest that prenatal sex steroid exposure (reflected in 2D:4D) affects not only birth weight, with statistically significant positive correlations revealed between 2D:4D R and the majority of examined parameters (body weight, BMI, waist and hip circumference) measured in children aged 6–13. These results indicate that prenatal estrogen exposure (higher 2D:4D) leads to increased weight-related parameters already in prepubertal children. Until now, some authors have rejected any relationship between those parameters in children and adolescents [16–18], but several papers have shown a significant effect of prenatal sex steroid exposure on e.g. BMI in children. Ranson et al. found a positive correlation between 2D:4D R and body weight, BMI, and waist circumference in girls (aged 8–12), but not in boys (*N* = 922 boys. 835 girls) [14]. In another study conducted by Klimek et al., feminized 2D:4D R values were associated with higher body weight and BMI in a group of 320 males aged 3–22 [15]. Manning et al. finding in adults showed that 2D:4D values (R & L) were positively related to measures of BMI in a very large sample (255,116 participants) [13]. Our findings in children study group are further evidence suggesting the positive relationship between 2D:4D and BMI is influenced prenatally.

The present study revealed positive correlations between 2D:4D and most postnatal body size parameters in both sexes. All of those correlations were found for the right hand, which is taken to be a better indicator of prenatal exposure to sex steroids [6]. Bagepally et al., who examined a large group (*N* = 1217) of male children, adolescents, and adults aged 13–40, reported that feminized 2D:4D ratios were significantly correlated with higher BMI values [9]. The longitudinal cohort study by Rich-Edwards et al. showed that low birth weight and high BMI increase the risk of type 2 diabetes and heart disease in adults [34]. Bazaes et al. came to similar conclusions, also finding that higher BMI values are characteristic of children with catch-up growth [40]. The present study did reveal that prenatal sex steroid exposure programs prenatal and postnatal body weight according to the sex of the child. In boys, the level of this exposure modified the relationship between birth weight and postnatal body weight. While in girls such interaction did not occur. Therefore, it should be concluded that in the girls, birth weight and body weight during progressive postnatal ontogenesis are additive correlates of the 2D: 4D L. Boys with lower birthweight tended to reach greater body weight during postnatal period in case of high prenatal oestrogen exposure (feminized 2D:4D L). When male foetus is strongly exposed to androgens its birth weight positively correlates with postnatal body weight. Due to the observed effect of the 2D 4D of the left hand on the relationship between birth weight and postnatal body weight, the obtained result should be treated with caution and this interaction should be verified in further studies.

This study identified a relationship between 2D:4D and hand grip strength, which is important in predicting the physical strength of children. While 2D:4D R was negatively correlated with right hand grip strength in boys and girls, in the case of 2D:4D L a similar relationship was found only in boys for both left and right hand grip strength. The previously cited study by Ranson et al., which analyzed the relationship between 2D:4D R and hand grip strength, also reported a negative correlation only in boys [14]. Similarly, Tomkinson and Tomkinson found that feminized 2D:4D R values were correlated with lower right hand grip strength in boys [41]. In a study involving girls, Peeters et al. used a very accurate X-ray method to determine 2D:4D R. In addition to hand grip strength, they investigated anthropometric parameters, including body weight and BMI, and selected aspects of physical fitness (speed, flexibility, etc.), but did not find any significant correlations between these variables [17]. Yet another study assessed elements of physical fitness (including speed, strength, agility, flexibility) along with hand grip strength in a group of 316 boys aged 7–13, but again no significant correlations with 2D:4D were found [42].

Additionally, the 2D:4D digit ratio in children can be considered an indicator of muscle strength in later life. Halil et al. reported that the higher the digit ratio, the lower the muscle strength among adults, regardless of sex. That relationship was found to continue in the elderly, directly influencing their quality of life [43]. The present investigations showed that the relationship between prenatal exposure to sex steroids and muscle strength manifests itself already in childhood. Furthermore. a longitudinal study has found that decreased hand grip strength may predict loss of cognitive function [44], which suggests that grip strength measurement can be used as a tool for monitoring cognitive status in aging populations.

It is necessary to develop additional indicators that would be helpful in identifying the lifelong consequences of impaired intrauterine development. Prenatal sex steroid exposure estimated by the 2D:4D digit ratio may be one of them as it is established in utero and remains invariant throughout ontogenesis. The present study suggests that sex steroid proportions seem to play a significant role in prenatal life, affecting the muscle strength. weight and proportions of the human body. New insights could be provided by investigating individual pathways of developing body parameters from the prenatal stage (2D:4D ratio) through childhood, adolescence, and adulthood.

## Supporting information

S1 Data (XLSX)
